# TyGIS: improved triglyceride-glucose index for the assessment of insulin sensitivity during pregnancy

**DOI:** 10.1186/s12933-022-01649-8

**Published:** 2022-10-18

**Authors:** Benedetta Salvatori, Tina Linder, Daniel Eppel, Micaela Morettini, Laura Burattini, Christian Göbl, Andrea Tura

**Affiliations:** 1 CNR Institute of Neuroscience, Corso Stati Uniti 4, 35127 Padua, Italy; 2 Department of Obstetrics and Gynaecology, Medical University of Vienna, 1090 Vienna, Austria; 3 Department of Information Engineering, Università Politecnica Delle Marche, 60131 Ancona, Italy

**Keywords:** Triglyceride-glucose index, Insulin resistance, Pregnancy, Gestational diabetes, Machine learning, Mathematical modelling

## Abstract

**Background:**

The triglyceride-glucose index (TyG) has been proposed as a surrogate marker of insulin resistance, which is a typical trait of pregnancy. However, very few studies analyzed TyG performance as marker of insulin resistance in pregnancy, and they were limited to insulin resistance assessment at fasting rather than in dynamic conditions, i.e., during an oral glucose tolerance test (OGTT), which allows more reliable assessment of the actual insulin sensitivity impairment. Thus, first aim of the study was exploring in pregnancy the relationships between TyG and OGTT-derived insulin sensitivity. In addition, we developed a new version of TyG, for improved performance as marker of insulin resistance in pregnancy.

**Methods:**

At early pregnancy, a cohort of 109 women underwent assessment of maternal biometry and blood tests at fasting, for measurements of several variables (visit 1). Subsequently (26 weeks of gestation) all visit 1 analyses were repeated (visit 2), and a subgroup of women (84 selected) received a 2 h-75 g OGTT (30, 60, 90, and 120 min sampling) with measurement of blood glucose, insulin and C-peptide for reliable assessment of insulin sensitivity (PREDIM index) and insulin secretion/beta-cell function. The dataset was randomly split into 70% training set and 30% test set, and by machine learning approach we identified the optimal model, with TyG included, showing the best relationship with PREDIM. For inclusion in the model, we considered only fasting variables, in agreement with TyG definition.

**Results:**

The relationship of TyG with PREDIM was weak. Conversely, the improved TyG, called TyGIS, (linear function of TyG, body weight, lean body mass percentage and fasting insulin) resulted much strongly related to PREDIM, in both training and test sets (R^2^ > 0.64, p < 0.0001). Bland–Altman analysis and equivalence test confirmed the good performance of TyGIS in terms of association with PREDIM. Different further analyses confirmed TyGIS superiority over TyG.

**Conclusions:**

We developed an improved version of TyG, as new surrogate marker of insulin sensitivity in pregnancy (TyGIS). Similarly to TyG, TyGIS relies only on fasting variables, but its performances are remarkably improved than those of TyG.

**Supplementary Information:**

The online version contains supplementary material available at 10.1186/s12933-022-01649-8.

## Introduction

Insulin resistance is a typical trait of pregnancy, even when not complicated by any endocrinological, metabolic or cardiovascular disorder [1–6]. Of note, when pregnancy is complicated by gestational diabetes mellitus (GDM), insulin resistance further deteriorates [7–10]. According to international guidelines, GDM can be diagnosed by fasting glycemia or by an oral glucose tolerance test (OGTT) [11]. With regard to the OGTT, GDM diagnosis requires collecting only the 60 min and the 120 min blood samples following glucose ingestion, and only glycemia needs to be assessed [11]. Thus, in routine clinical practice, the OGTT includes only two time samples in addition to the fasting sample, and with no measurement of insulin or C-peptide. In fact, more complete OGTT studies are currently limited to clinical trials rather than the clinical routine. However, the information of the simple diagnostic OGTT do not allow assessment of insulin resistance, which requires insulin measurement in addition to glucose and takes advantage from possible collection of more blood samples rather than the 60 and 120 min samples only [12–17]. Nonetheless, the assessment of insulin action may be clinically relevant in GDM and even in pregnant women with overweight, obesity or with signs of dysglycemia (though in the absence of overt GDM), since the degree of insulin resistance can drive specific therapeutic intervention and care [18, 19]. In addition, though there are still controversies about optimal timing for metabolic assessment in pregnancy [20, 21], the assessment at early pregnancy (i.e., before 24 weeks of gestation) of the insulin resistance level (and, possibly, other metabolic parameters) may allow prediction of GDM risk and severity of the disease at later stage. Thus, the problem arises on how to assess insulin resistance or sensitivity without the OGTT or even more complicated tests, such as the hyperinsulinemic-euglycemic clamp, which are hardly feasible in the clinical practice (especially in pregnant women).

There is an increasing interest in the triglyceride-glucose index (TyG) [22], as mirrored by the remarkably growing number of articles dealing with TyG in the recent years. Especially, in the 2019–2021 period the number of pertinent articles has increased each year by about the 50% compared to the previous year, and at mid-2022 the number of articles has already reached that of the whole 2020 (source: PubMed). This is likely due to the relevant properties of TyG, despite its simple formulation, as it only relies on fasting triglycerides and glucose [22]. The importance of TyG has in fact been summarized in several review studies, which have shown TyG ability to predict risk for cardiovascular diseases, type 2 diabetes and GDM as well [23–32]. In addition, TyG has been proposed in several investigations as surrogate marker of insulin resistance [24, 33, 34]. TyG as surrogate of insulin resistance appears in fact the original reason for its introduction in the scientific community [22, 35]. Notably, insulin resistance is a relevant aspect of the metabolic syndrome, and, as such, an important risk factor for cardiovascular diseases or diabetes [36–46]. However, studies on TyG potential and relevance in pregnancy are still relatively scarce [30, 47–55]. Furthermore, studies on TyG performance as surrogate marker of insulin resistance in pregnancy are even rarer [47, 51], and limited to the assessment of insulin resistance at fasting rather than in dynamic conditions (i.e., during a glucose or meal challenge), which allow more reliable assessment of the actual degree of whole body insulin resistance. On the other hand, in a previous study from our research group we found that in pregnant women fasting triglycerides are associated with insulin resistance derived from an OGTT [56]. However, we did not investigate the relationships between OGTT-derived insulin resistance and TyG.

Thus, one aim of the present study was to explore OGTT-derived insulin resistance and TyG relationships in pregnancy. To our knowledge, in pregnancy no previous study analyzed the performance of TyG as marker of insulin resistance derived by a metabolic test, such as the OGTT. In addition, and most importantly, we aimed to develop a new version of TyG for improved performance as marker of OGTT-derived insulin resistance in pregnancy, spanning from normal glucose tolerance to overt GDM. This was accomplished by exploiting advanced methodologies based on machine learning techniques. Notably, to ensure simplicity of the improved TyG, and hence wide applicability in the clinical context, the possible factors assessed as possible complements of TyG for OGTT-derived insulin sensitivity prediction were restricted to those stemming from fasting measures or general subjects’ characteristics.

## Methods

### Study design, participants and measures

Between 2015 and 2020, 109 pregnant women were recruited among all women attending the pregnancy outpatient department at the Medical University of Vienna. Women after bariatric surgery, with preconceptional (type 1 or type 2) diabetes or other endocrine disorders, HIV, hepatic infection or malignant tumors were excluded. At early pregnancy (visit 1, median gestational age: 15.9 weeks, interquartile range, IQR: 13.4 to 18.9) all women underwent assessment of maternal biometry and blood tests at fasting, for measurements of total cholesterol, HDL-cholesterol, LDL-cholesterol, hemoglobin (Hb), glycated hemoglobin (HbA1c), aspartate aminotransferase (ASAT), alanine aminotransferase (ALAT), gamma glutamyltransferase (GGT), albumin, creatinine, C-reactive protein, platelet count, glucose, insulin and C-peptide.

At a median gestational age of 26.0 weeks (IQR: 25.3 to 27.1) all analyses were repeated, and a subgroup of women received a 2 h-75 g OGTT to assess dynamic parameters of glucose metabolism (visit 2). After an overnight fast, women ingested a solution containing 75 g of glucose and venous blood samples were taken at fasting and for 120 min (at 30, 60, 90, and 120 min) for blood glucose, insulin and C-peptide measurements. GDM was diagnosed according to the International Association of the Diabetes and Pregnancy Study Groups recommendations (in some women presence of GDM was verified by glucose self-monitoring in accordance with Austrian national guidelines) [57]. Maternal and neonatal parameters and outcomes were also recorded. Calculations of age and sex adjusted percentiles of newborns’ birth weight were based on international anthropometric standards, whereby LGA (large for gestational age) was defined as birth weight above the 90th percentile [58]. In the present analysis, following exclusion of some women due to OGTT data not suitable for modelling and machine learning analysis (*e.g.*, incomplete OGTT data or presence of outliers), we ended up with a group of 84 women, having glucose, insulin and C-peptide measurement at every time sample of the OGTT, as well as measurement of all variables indicated above. Data of a subset of the participants included in this work were previously published [56], whereas other participants derived from an internal cohort, collected with comparable study design. The study was approved by the Ethics Committee of the Medical University of Vienna and performed in accordance with the most recent version of the Declaration of Helsinki. All participants gave written informed consent.

### Laboratory analysis details

All laboratory parameters were measured according to the standard methods at the Department of Medical and Chemical Laboratory Diagnostics (http://www.kimcl.at). As regards the OGTT variables, plasma glucose was measured by the hexokinase method (coefficient of variation (C.V.): 1.3%, 101 mg/dL), whereas insulin (C.V. from 4 to 7%, 2 µU/mL) and C-peptide (C.V. from 3 to 4%, 0.08 ng/mL) were measured by luminescence immunoassays. HbA1c was assessed by high-performance liquid chromatography using Variant II, Bio-Rad, International Federation of Clinical Chemistry (IFCC) standardized and Diabetes Control and Complications Trial (DCCT)-aligned with C.V. of 1.8% (HbA1c = 5.6%).

### Calculation of insulin sensitivity and beta-cell function

At visit 2 (when OGTT data were available), total body insulin sensitivity was assessed by the PREDIM (predicted M) index, which has shown particularly good performances against the gold-standard insulin sensitivity index derived from the hyperinsulinemic-euglycemic clamp technique [59]. Specific parameters describing different aspects of insulin secretion and beta-cell function were assessed from C-peptide according to mathematical modelling [60, 61]. Briefly, we assessed basal insulin secretion rate (BSR) and total insulin secretion (TIS), glucose sensitivity (G-sens, representing the mean slope of the dose response function describing insulin release for the absolute glucose levels), rate sensitivity (R-sens, representing early insulin release for the rate of change of glucose) and potentiation factor (PFR, representing the ratio of the insulin secretion potentiation at the end *vs.* the beginning of the OGTT).

### Determination of an improved version of the triglyceride-glucose index

*The machine learning approach*: at visit 2, we exploited the traditional TyG values and other data measured at the time of the OGTT to identify by machine learning approach an improved version of TyG, which can accurately predict the OGTT-derived insulin sensitivity as assessed by PREDIM. We named the new index TyGIS (where IS stands for insulin sensitivity). Of note, the clinical variables that we analyzed for possible inclusion in the TyGIS formulation were only those measured at fasting, rather than during the dynamic conditions of the OGTT. Indeed, we aimed to preserve the basic concept behind TyG, this being simplicity of calculation due to need for variables measured at fasting only, thus not requiring glucose challenge or other metabolic tests. It is also worth clarifying that TyG is a possible marker of insulin resistance, whereas TyGIS is built as a marker of insulin sensitivity, for direct comparison with PREDIM (the latter being an index of insulin sensitivity rather than resistance).

One key factor towards the possible clinical application of machine learning models is the easy interpretability [62]. To this purpose, we aimed to develop a model described by a simple algebraic equation. To reach the final model formulation, multivariate polynomial regression was employed using the L2-regularized (also known as Ridge) Support Vector Machine approach (SVM) [63]. Nonetheless, other model building strategies were tested that showed superior performance over ordinary least squares regression in previous studies [64], such as L1-regularized (or LASSO, i.e., least absolute shrinkage and selection operator) SVM [63] and robust linear regression [65]. All models were implemented in MATLAB (version 2020a) and related Statistics and Machine Learning toolbox (MathWorks, Inc., USA). More details about our machine learning approach are reported in the following paragraphs.

*Feature selection procedure*: from the original clinical data, we initially considered several variables (features) for possible inclusion in the formulation of TyGIS. The value of each analyzed feature was that at the time of the OGTT (i.e., visit 2), except obviously for pre-pregnancy weight. The first considered feature was the traditional TyG, whose definition is $TyG=ln[fasting\, triglycerides \cdot fasting\, glucose /2]$, with both triglycerides and glucose in mg/dL [22]. Since TyGIS is intended being an improved TyG, our procedure forced TyG to be included among the selected features. We also tested possible inclusion of triglycerides and glucose separately. The other features considered in this analysis were age, height, pre-pregnancy body weight, body weight, body mass index (BMI), body surface area (BSA), lean body mass (LBM), total pregnancy number, parity, week of gestation, multiple pregnancy (*e.g.*, twin pregnancy), GDM condition, total cholesterol, HDL-cholesterol, LDL-cholesterol, Hb, HbA1c, ASAT, ALAT, GGT, albumin, creatinine, C-reactive protein, platelet count, fasting insulin, fasting C-peptide. The body mass index was computed as traditionally: $BMI=body\, weight/{height}^{2}$; body surface area was $BSA=0.164\cdot { body\, weight}^{0.515}\cdot {height}^{0.422}$ [66]; lean body mass was  $LBM=0.296\cdot body\, weight +41.813\cdot height-43.293$ [67] (with body weight in kg and height in meters in all formulas).

The dataset of the studied 84 women was split into 70% training set and 30% test set, according to common practice [68], thus obtaining a training set of 59 women and a test set of 25. While the training set was used to implement the model, the test set was used only after the final model was determined, thus obtaining an independent evaluation of the generalization capability of the new index formulation (i.e., of TyGIS performance in predicting PREDIM). Standardization of each feature was carried out on the training set data, yielding zero mean and unit variance for each feature, to cope with the dependence of the implemented learning algorithms on the feature scales [69]. This phase of the procedure yielded the selection of 46 subsets as inputs for the following phases in model formulation (see Additional file 1 for details).

**Fig. 1:**
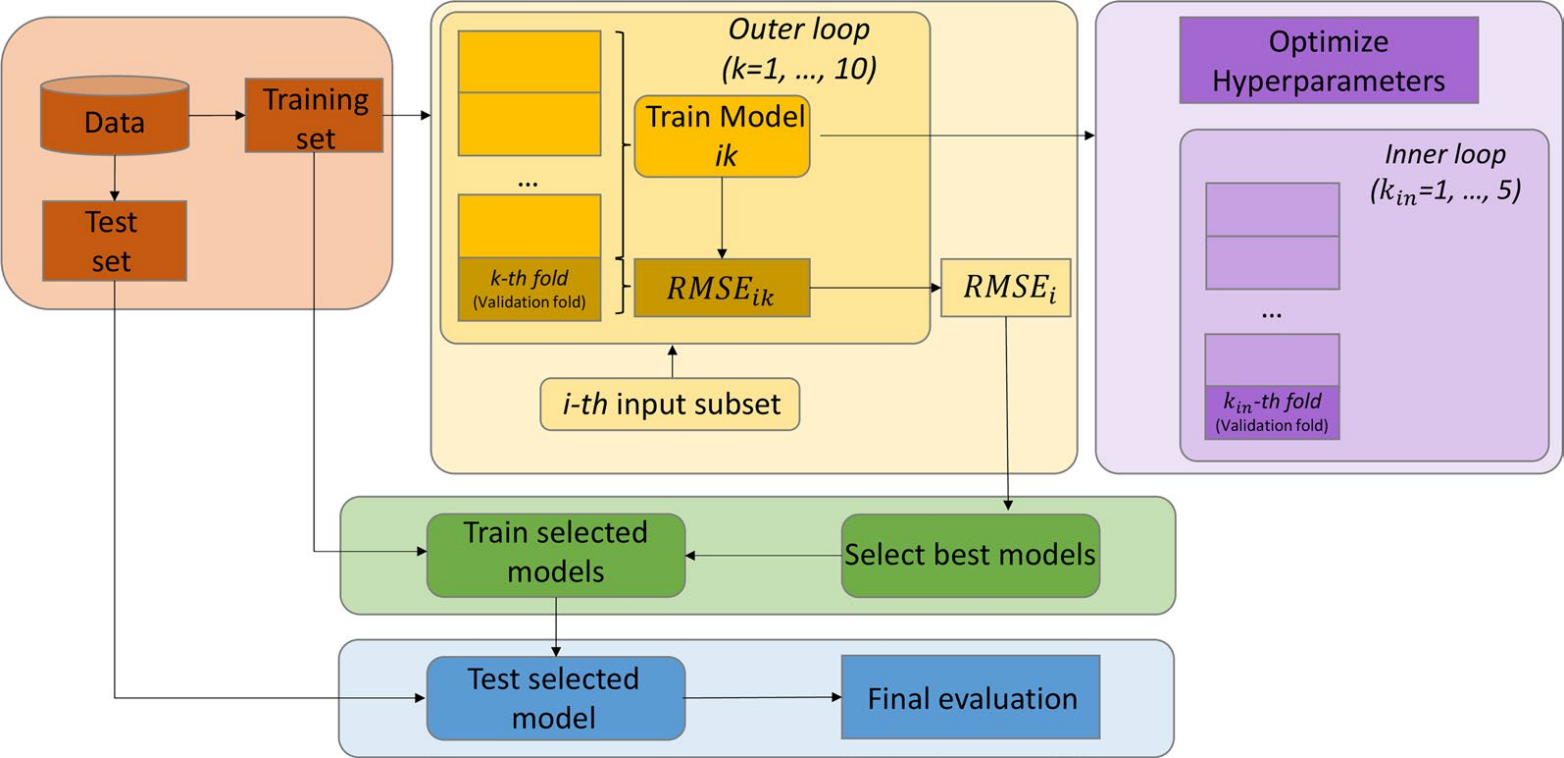
Framework for generation and selection of the regularized SVM regression model. The yellow and purple blocks outline the implemented nested cross-validation procedure

*Final model generation, selection and application*: for the final model generation and selection, a nested CV technique was implemented, consisting of an outer and an inner loop (Fig. 1). For ensuring low features to sample size ratio [70], in the selection of the final model both the root mean squared error (RMSE) and the Bayesian information criterion (BIC) [71] values were considered, preferring models with fewer predictors that still achieved good performance. Once the best input was selected (that is, the final model determined), such model was re-trained on the training set, and its generalization capability was finally evaluated on the test set. Further details on this phase of the procedure are reported in Additional file 1.

It has to be emphasized that the final model for TyGIS calculation was determined at visit 2, when PREDIM was available. However, TyGIS was then computed also at visit 1. Indeed, at visit 2 we tested the association between TyGIS and PREDIM, computed only at visit 2 as it requires OGTT data (only available at visit 2). On the other hand, TyGIS computation at visit 1 allowed investigating its ability to predict insulin sensitivity at later pregnancy period (i.e., PREDIM at visit 2).

### Statistical analysis

Linear regression analysis was performed between PREDIM and the new TyG index, TyGIS, as well as between PREDIM, TyGIS, TyG and the model-derived parameters of insulin secretion and beta-cell function (BSR, TIS, G-sens, R-sens, PFR). PREDIM and TyGIS were also compared by Bland–Altman plot. In addition, test of equivalence was also used to compare PREDIM and TyGIS [59]. In that analysis, we identified how small the equivalence limit parameter (representing the tolerance of the test) could be while maintaining the significance of the test (i.e., p-value just slightly lower than 0.05) [59].

Comparison of one variable between two groups was performed by the Welch’s t-test, to account for typically different variable variances in the groups. For comparison among three groups, we used analysis of variance (ANOVA), and Dunnett Tukey Kramer post-hoc test for comparison in pairs of groups. Fisher’s exact test was used for group comparison of categorical variables. Lastly, correlation between TyGIS at the time of the first visit (visit 1) and PREDIM at visit 2 was evaluated. Similarly, a logistic regression analysis was performed to investigate whether TyGIS at visit 1 can be predictive of non-normal glucose tolerance [72] or GDM condition at visit 2. Some of the indicated analyses were repeated for the traditional TyG.

Continuous variables were summarized by mean ± standard deviation (SD) or median and IQR (depending on variable distribution, according to Shapiro–Wilk test). Categorical variables were summarized by counts and percentages. For continuous variables, log_e_ transformation was applied in case of skewed distribution. A two-sided p < 0.05 was considered statistically significant. Due to the explorative character of this observational study, we used no adjustment for multiple statistical testing if not otherwise indicated. Statistical analysis was performed with R (V4.0.2) and contributing packages.

## Results

### Features and OGTT parameters

Table 1 reports the features value in the studied women divided for glucose tolerance, i.e., normal glucose tolerance (NGT) and non-NGT (GDM or impaired glucose regulation), or divided for BMI, i.e., Lean and Overweight (BMI ≥ 25 kg/m^2^), in both cases according to the features value at visit 2.

**Table 1:** Feature values in pregnant women divided for glucose tolerance or for body mass index (BMI)

Feature	NGT	Non-NGT	Lean	Overweight/Obese
*N*	72	12	23	61
TyG index (unitless)	8.84 ± 0.36	9.12 ± 0.42^a^	8.69 ± 0.35	8.96 ± 0.37^f^
Age (years)	30.1 ± 4.9	32.8 ± 6.0	30.3 ± 4.8	30.5 ± 5.3
Pre-pregn. body weight (kg)	63.50 [13.50]	76.00 [33.85]^a^	59.00 [8.75]	70.00 [19.13]^h^
Body weight (kg)	72.95 [15.60]	81.20 [33.00]^a^	62.00 [7.38]	77.00 [14.64]^h^
Height (m)	1.66 ± 0.06	1.63 ± 0.06	1.67 ± 0.07	1.65 ± 0.06
BMI (kg/m^2^)	26.51 [4.35]	29.80 [11.51]^d^	22.46 [1.68]	28.07 [5.54]^h^
BSA (m^2^)	1.88 [0.25]	2.02 [0.35]	1.75 [0.13]	1.93 [0.23]^h^
LBM (%)	48.81 ± 5.42	51.75 ± 7.71	45.90 ± 4.64	50.49 ± 5.78
Total pregnancy no. (counts)	2.00 [2.00]	2.00 [2.00]	2.00 [2.00]	2.00 [2.00]
Parity (counts)	0.00 [1.00]	0.00 [0.50]	0.00 [1.00]	0.00 [1.00]
Multiple pregn. (counts, perc.)	8 (11.1%)	0 (0.0%)	1 (4.3%)	7 (11.5%)
Week of gestation (weeks)	26.00 [1.86]	25.86 [1.28]	25.57 [1.39]	26.14 [1.85]
GDM (counts, perc.)	0 (0.0%)	9 (75.0%)^d^	1 (4.3%)	8 (13.1%)
Fasting glucose (mmol/L)	4.27 [0.56]	4.91 [0.56]^b^	4.16 [0.49]	4.44 [0.72]^e^
Fasting insulin (pmol/L)	51.30 [44.49]	86.97 [64.17]^a^	45.36 [48.38]	57.06 [47.72]^e^
Fasting C-peptide (nmol/L)	0.63 [0.28]	1.03 [0.46]^b^	0.60 [0.26]	0.73 [0.40]^f^
Hb (g/L)	112.75 ± 9.89	118.17 ± 10.68	113.65 ± 11.83	113.48 ± 9.51
HbA1c (%)	4.79 ± 0.30	5.3 ± 0.35^c^	4.71 ± 0.34	4.92 ± 0.34^e^
Platelet count (U/L)	242.50 [54.50]	279.50 [58.50]	243.00 [37.00]	250.00 [80.00]
C-reactive protein (mg/L)	0.40 [0.58]	0.77 [0.47]	0.29 [0.58]	0.47 [0.61]
Albumin (g/L)	39.68 ± 2.32	39.93 ± 2.47	39.73 ± 2.55	39.71 ± 2.26
Creatinine (µmol/L)	43.77 [8.84]	41.56 [11.94]	42.44 [10.17]	43.33 [8.84]
Triglycerides (mmol/L)	1.99 [0.95]	2.36 [1.08]	1.85 [0.80]	2.10 [1.05]^f^
Cholesterol (mmol/L)	6.53 ± 1.27	6.28 ± 1.22	6.69 ± 1.38	6.42 ± 1.21
HDL (mmol/L)	1.89 [0.80]	1.70 [0.56]	2.07 [0.87]	1.84 [0.85]
LDL (mmol/L)	3.54 ± 1.10	3.37 ± 0.99	3.76 ± 1.22	3.42 ± 1.01
ASAT (U/L)	20.00 [8.00]	20.50 [6.00]	22.00 [8.00]	20.00 [7.00]
ALAT (U/L)	14.00 [6.75]	15.50 [14.00]	14.00 [5.00]	14.00 [10.00]
GGT (U/L)	8.00 [4.00]	11.50 [21.00]	9.00 [4.00]	8.00 [5.50]

Data for continuous variables are reported as mean ± SD or median [IQR] as appropriate, and as counts and percentages for categorical variables. Except for pre-pregnancy body weight, the values of all anthropometric and clinical features refer to visit 2. Accordingly, stratification in NGT and non-NGT, as well as Lean and Obese/Overweight, refers to visit 2

*NGT* normal glucose tolerance, *Lean* BMI < 25 kg/m^2^, *Overweight/Obese* otherwise

^a^p < 0.05 for NGT and Non-NGT

^b^p < 0.01 for NGT and Non-NGT

^c^p < 0.001 for NGT and Non-NGT

^d^p < 0.0001 for NGT and Non-NGT

^e^p < 0.05 for Lean and Overweight/Obese

^f^p < 0.01 for Lean and Overweight/Obese

^g^p < 0.001 for Lean and Overweight/Obese

^h^p < 0.0001 for Lean and Overweight/Obese

In Table 2, we reported the values of insulin sensitivity (PREDIM), insulin secretion (BSR, TIS) and beta-cell function (G-sens, R-sens, PFR), as derived by the OGTT.

**Table 2:** OGTT parameters in women divided for glucose tolerance or for body mass index (BMI)

OGTT parameter	NGT	Non-NGT	Lean	Overweight/Obese
*N*	72	12	23	61
Insulin sensitivity				
PREDIM (mg kg min^−1^)	5.89 [2.34]	2.89 [1.01]^d^	6.49 [3.57]	4.88 [2.54]^g^
Insulin secretion				
BSR (pmol min^–1^ m^–2^)	84.12 [39.63]	136.20 [48.33]^b^	77.84 [36.27]	99.68 [47.85]^f^
TIS (nmol m^–2^)	46.80 [19.42]	69.06 [16.96]^c^	44.92 [16.85]	54.16 [22.39]
Beta-cell function				
G-sens (pmol min^–1^ m^–2^ mM^–1^)	140.24 [81.51]	89.92 [31.27]^d^	154.55 [106.32]	122.33 [59.01]
R-sens (pmol m^–2^ mM^–1^)	1028.41 [1313.86]	706.17 [705.18]	716.84 [1727.23]	1028.36 [974.72]
PFR (unitless)	1.35 [0.49]	1.42 [0.71]	1.35 [0.40]	1.37 [0.61]

Data are reported as median [IQR]. The values of the parameters refer to visit 2. Accordingly, stratification in NGT and non-NGT, as well as Lean and Obese/Overweight, refers to visit 2

*NGT* normal glucose tolerance, *Lean* BMI < 25 kg/m^2^, *Overweight/Obese* otherwise

^a^p < 0.05 for NGT and Non-NGT

^b^p < 0.01 for NGT and Non-NGT

^c^p < 0.001 for NGT and Non-NGT

^d^p < 0.0001 for NGT and Non-NGT

^e^p < 0.05 for Lean and Overweight/Obese

^f^p < 0.01 for Lean and Overweight/Obese

^g^p < 0.001 for Lean and Overweight/Obese

^h^p < 0.0001 for Lean and Overweight/Obese

Of note, with regard to the features available in the whole dataset (*N* = 109), both at visit 1 and visit 2 there was no difference between the whole dataset and the studied women (*N* = 84) dataset, in any of the features (details not shown).

### Performance in training set

The model selected was the one with minimum BIC value, which also achieved low RMSE on the CV validation folds, and it corresponded to a linear model with four predictors: TyG, body weight, fasting insulin and LBM. With this model, the new index for insulin sensitivity prediction, called TyGIS, was calculated as follows:

1 $$TyGIS=\,A \times TyG+B \times Body\, Weight+C \times Fasting\, Insulin+D \times LBM+E$$

Coefficient values are:

$$A=-0.4670326,B=-0.1219702,C=-0.0226746,D=0.2214735,E=9.7092789$$

Of note, though the Eq. (1) was obtained from the analysis of the normalized features version, for easier usability we finally expressed Eq. (1) with non-normalized features. Units of selected features are kg for Body Weight, pmol/L for Fasting Insulin, and % for LBM; TyG is unitless, but it has to be calculated with triglycerides and glucose in mg/dL, as in its original formulation [22].

**Fig. 2:**
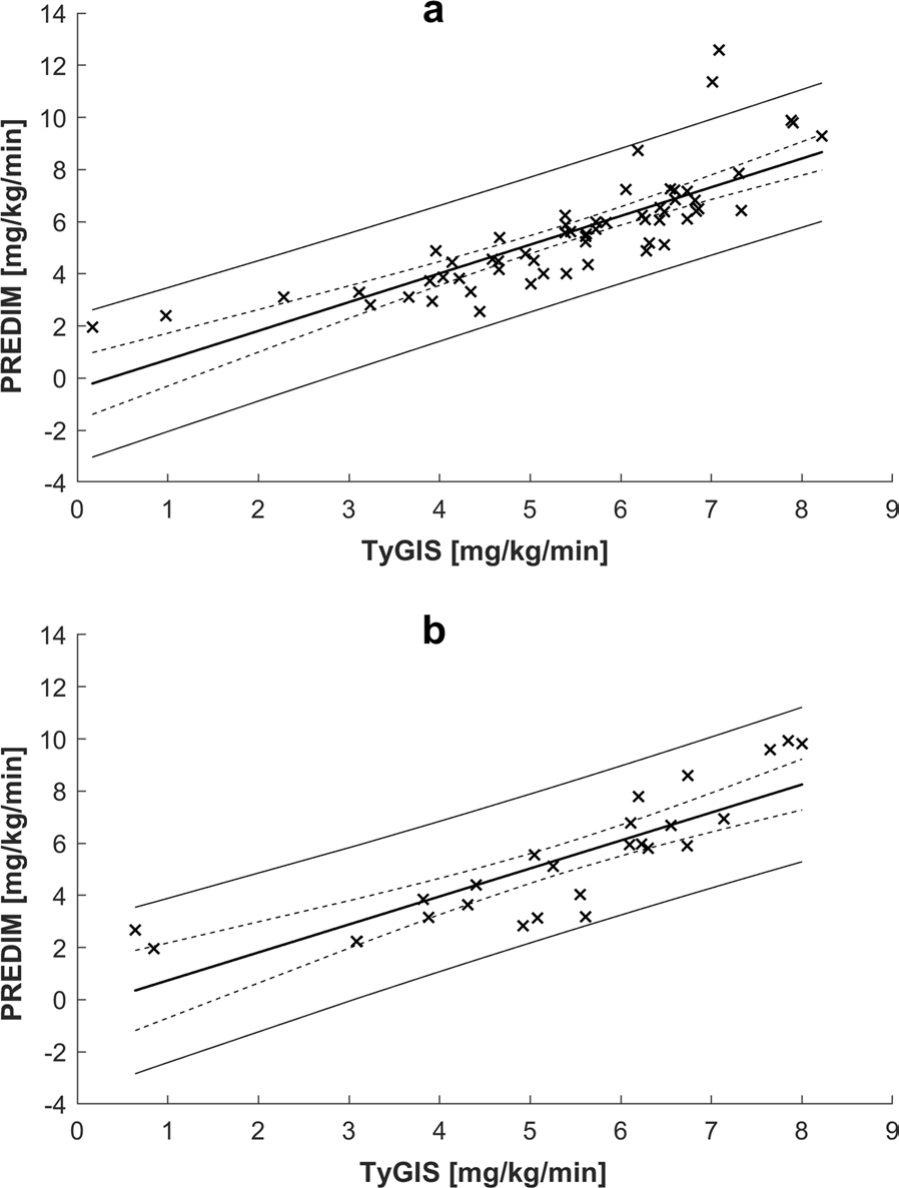
Linear regression plot in the training set (**a**) and the test set (**b**). Regression equations (bold solid line) are $y=1.102x-0.388$, R^2^ = 0.649, p < 0.0001 (**a**), and $y=1.073x-0.335$, R^2^ = 0.700, p < 0.0001 (**b**). The 95% confidence and prediction intervals are also reported (dashed and solid lines, respectively)

**Fig. 3:**
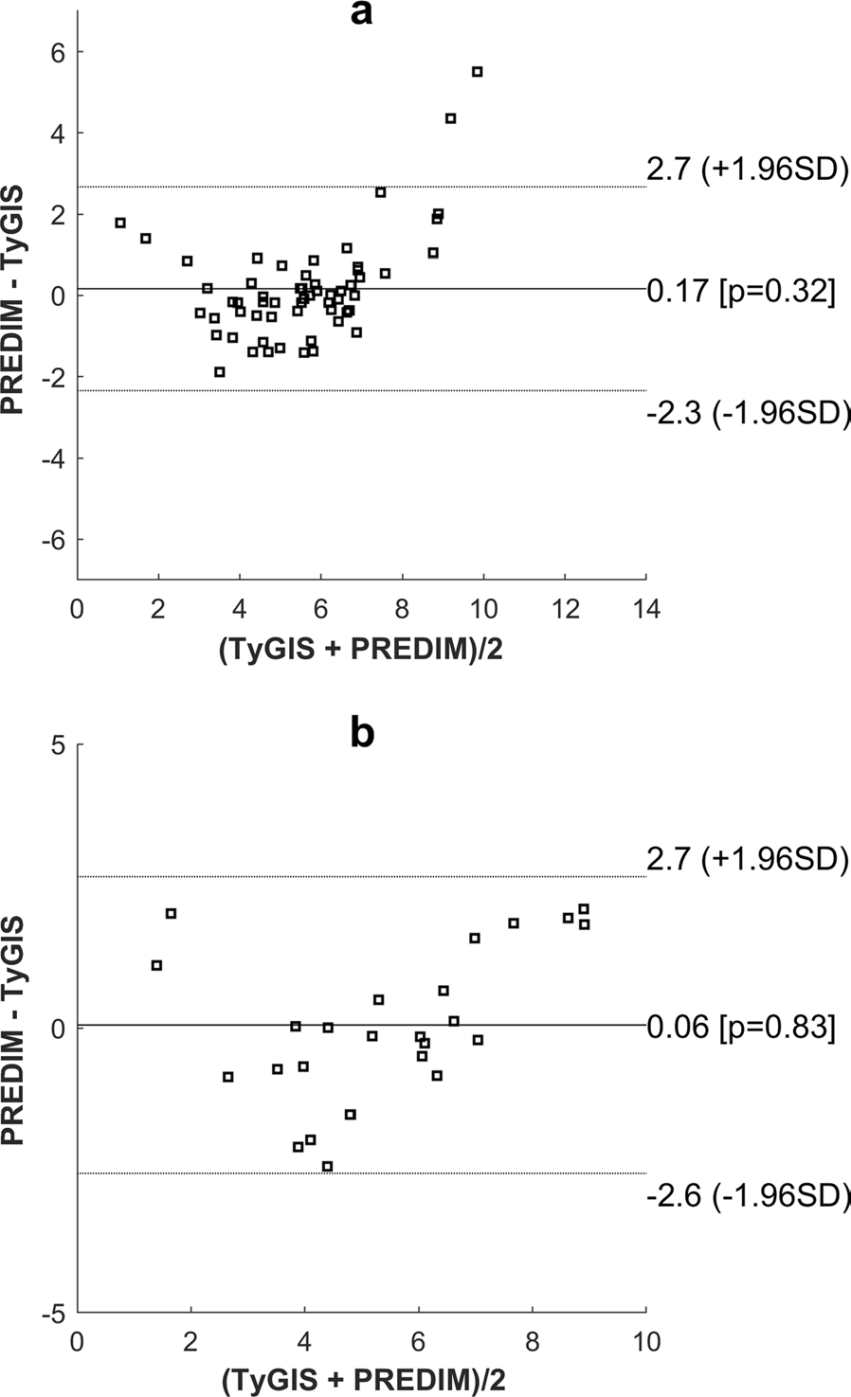
Bland–Altman plot in the training set (**a)** and the test set (**b**)

In the training set, when comparing TyGIS to PREDIM by linear regression analysis, we found adjusted R^2^ of 0.642, p < 0.0001 (Fig. 2a). In contrast, the relationship between TyG and PREDIM was not significant (p = 0.21). The Bland–Altman plot showed that only 2 out of 59 observations were outside the limits of agreement (Fig. 3a), which corresponded to the extreme PREDIM values, this suggesting possible lower agreement on higher insulin sensitivity. According to the equivalence test, PREDIM and TyGIS values were virtually identical up to a tolerance (equivalence limit parameter) equal to only 8.0% of mean PREDIM, with mean difference between the two indices of 0.166 (p < 0.05).

### Performance in test set

When applying Eq. (1) to the test set to analyze the proposed model generalization capability, we again found good relationship between TyGIS and PREDIM, with adjusted R^2^ = 0.687, p < 0.0001, by linear regression analysis (Fig. 2b). When assessing TyG and PREDIM relationship, it was still significant, but with R^2^ considerably lower (adjusted R^2^ = 0.425, p < 0.001). For TyGIS, Bland–Altman plot did not detect any observation outside the limits of agreement (Fig. 3b). According to the equivalence test, PREDIM and TyGIS values were similar up to a tolerance equal to 9.2% of mean PREDIM, showing a mean difference equal to 0.058 (p < 0.05).

### Subgroups discrimination

**Fig. 4:**
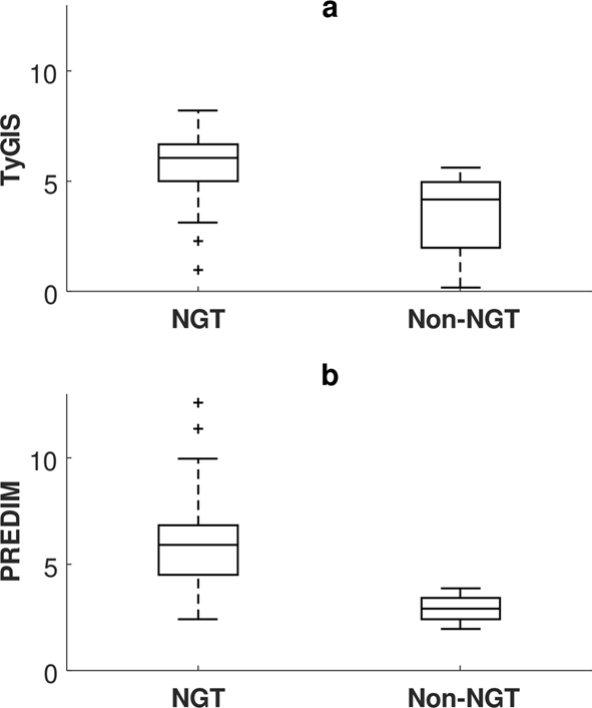
Boxplot for TyGIS (**a**) and PREDIM (**b**) in the subsets of subjects stratified by glucose tolerance

**Fig. 5:**
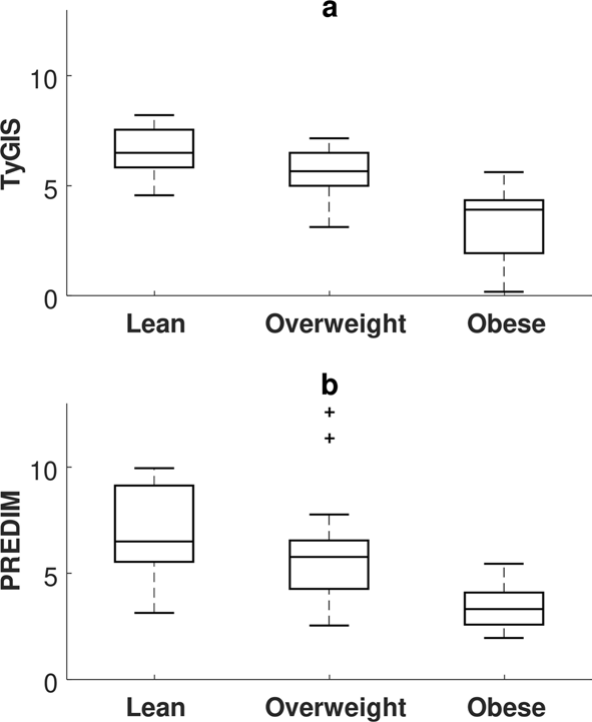
Boxplot for TyGIS (**a**) and PREDIM (**b**) in the subsets of subjects stratified by BMI

When comparing the NGT to the non-NGT subgroup (i.e., including women with GDM, or with impaired fasting glucose or impaired glucose tolerance [72]), both TyGIS and PREDIM showed significant difference between the two subgroups (p < 0.002 and p < 0.0001, respectively; see Fig. 4), whereas the traditional TyG showed borderline p-value (p = 0.046). In lean and overweight subgroups, TyGIS and PREDIM again consistently showed significant difference (p < 0.0001 and p < 0.001, respectively). TyG showed as well significant difference, but with less marked p-value (p < 0.01). In addition, with women stratified into normal weight, simple overweight (BMI ≥ 25 and < 30 kg/m^2^) and obesity (BMI ≥ 30 kg/m^2^), both TyGIS and PREDIM revealed progressive significant reduction for increasing levels of overweight (Fig. 5), with p < 0.01 and p < 0.05 for lean-overweight comparison, p < 0.0001 and p < 0.001 for overweight-obese comparison, respectively, and p < 0.0001 for both indices for lean-obese comparison. In contrast, the TyG did not show significant difference either in lean-overweight or in overweight-obesity comparison (p = 0.06 and p = 0.25, respectively).

### Relationships between insulin sensitivity and insulin secretion/beta-cell function

Results of regression analysis in all studied women (*N* = 84) between model-derived insulin secretion/beta-cell function parameters and insulin sensitivity (PREDIM), as well as TyGIS, and TyG, are summarized in Table 3.

**Table 3:** Adjusted R^2^ in regression analysis between insulin secretion/beta-cell function parameters (BSR, TIS, G-sens, R-sens, PFR) and PREDIM, TyGIS and TyG

	BSR	TIS	G-sens	R-sens	PFR
PREDIM	0.402^d^	0.221^d^	N.S.	N.S.	0.042^a^
TyGIS	0.642^d^	0.143^c^	N.S.	N.S.	N.S.
TyG	0.290^d^	0.130^c^	N.S.	N.S.	N.S.

This analysis was performed over all studied women (*N* = 84), with values of the parameters calculated at visit 2. The relationship of BSR and TIS with TyG was direct, whereas with PREDIM and TyGIS was inverse

*N.S*. non-significant

^a^p < 0.05

^b^p < 0.01

^c^p < 0.001

^d^p < 0.0001

It can be appreciated that relationships between insulin secretion/beta-cell function parameters and PREDIM, TyGIS, and TyG were typically weak or even absent. From this point of view, PREDIM, TyGIS, and TyG showed consistent results.

### Prediction performance

In all studied women, the correlation between TyGIS computed at visit 1 and PREDIM at visit 2 was relatively strong, yielding an adjusted R^2^ of 0.513, p < 0.0001. Instead, the relationship between TyG at visit 1 and PREDIM at visit 2 was very weak (virtually absent), with an adjusted R^2^ of 0.083 (p < 0.01).

Regarding the ability of TyGIS at visit 1 to predict non-NGT or specifically GDM (9 of the 12 non-NGT) at visit 2, logistic regression showed significant Odds Ratio (OR) values. For non-NGT prediction, OR was equal to 0.499 (95% Confidence Intervals, CI 0.335–0.743, p < 0.001), i.e., the higher the TyGIS, the lower the probability of later non-NGT condition. TyG at visit 1 proved as well to be a predictor of non-NGT condition, but with borderline p-value (OR = 9.116, 95% CI 1.854–44.817, p < 0.05). Similar results were found for the specific GDM condition, with again marked prediction performance for TyGIS (OR = 0.476, 95% CI 0.307–0.736, p < 0.001) and only borderline p-value for TyG (OR = 19.607, 95% CI 2.814–136.604, p < 0.05). Of note, since TyGIS has been built as marker of insulin sensitivity (direct predictor of PREDIM), whereas TyG is a possible marker of insulin resistance, OR value was lower than 1 for TyGIS and higher than 1 for TyG.

## Discussion

TyG has been shown to be a surrogate marker of insulin resistance [24, 33, 34]. In this study, we developed an improved version of TyG, for insulin sensitivity assessment in pregnancy. This is particularly important in this population, since impairment in insulin sensitivity is a common trait of pregnancy, even when normal. The new index, called TyGIS, was based on TyG complemented to other parameters. To maintain the simplicity of TyG (likely one of the reasons for its success) we only considered basic patient’s clinical characteristics and variables measured at fasting, thus not requiring metabolic tests such as the OGTT. This choice was also driven by the notion that the OGTT is not always and ubiquitously performed in pregnancy, and when performed the current clinical routine accomplishes a simple three samples, glucose only OGTT, for possible GDM diagnosis. Instead, in our study a more complete OGTT was exploited to compute a reliable parameter of insulin sensitivity (PREDIM), fully validated against the hyperinsulinemic-euglycemic clamp [59]. Another benefit of our dataset was the heterogeneity of the population, since women had glucose tolerance spanning from normal to GDM, and BMI from lean to overweight or even obese. In addition, the OGTT included glucose, insulin and C-peptide at each time sample, such complete OGTT allowing deep characterization of the TyGIS performance.

We found that TyGIS is a remarkably better surrogate marker than the traditional TyG for insulin sensitivity assessment in pregnancy. Indeed, TyGIS is much strongly associated with the OGTT-derived insulin sensitivity (PREDIM). It also has to be noted that TyGIS was developed as an index of insulin sensitivity, although TyG is an index of insulin resistance. This was done for more direct comparison of TyGIS to its reference, PREDIM, which is an index of insulin sensitivity rather than resistance. We also found that, at difference with TyG, TyGIS agrees with PREDIM as regards determination of insulin sensitivity differences among several subgroups of patients, stratified according to glucose tolerance or BMI. Furthermore, TyGIS calculated at early pregnancy (patients’ visit 1) was good predictor of PREDIM and of abnormal glucose tolerance at later pregnancy stage (second trimester, visit 2), again showing superiority compared to TyG.

It was previously reported that 4.9 mg∙kg^−1^∙min^−1^, as derived by the clamp (the *M* parameter), can be assumed as cut-off for insulin resistance [73]. PREDIM was developed as predictor of clamp-derived *M* parameter, with the same units and expected range of variation [59]. Similarly, TyGIS was developed as predictor of PREDIM. Thus, the same cut-off value can be reasonably assumed for TyGIS, for determination of insulin resistance. Interestingly, both PREDIM and TyGIS showed several values below that cut-off even in the absence of GDM (details not shown), this being reasonable for women during pregnancy. This result further indicates the reliability of our study findings.

In our study, we also analyzed relationships with model-based parameters of insulin secretion and beta-cell function, this being an important aspect for detailed testing of the new index performance. All indices (TyG, TyGIS and PREDIM) agreed in terms of correlation with insulin secretion parameters (BSR and TIS). All three indices also agreed in showing substantial lack of correlation with the beta-cell function parameters (G-sens, R-sens, PFR). For R-sens and PFR, it has to be noted that in the general population the degree of relationship with insulin sensitivity remains partly unclear, whereas several studies showed that G-sens (the most relevant beta-cell function model parameter) is typically unrelated to insulin sensitivity (this is an advantage, since it implies that there is no need to correct for the insulin sensitivity level for appropriate interpretation [74–79]). Thus, these analyses based on insulin secretion and beta-cell function parameters further proved the reliability of the TyGIS formulation.

In the TyGIS formula, one may expect the presence of BMI. Instead, anthropometry contribution to the new index was provided by simple body weight (BW), as well as lean body mass percentage (LBM, computed by an empirical formula [67]). Nonetheless, if BW and LBM are replaced by BMI, the performance of the new index deteriorates only slightly (details not shown). The fact that the BMI role resulted to some extent less significant than that of BW and LBM may be due to the reason that in pregnancy the relationship between insulin resistance and BMI may be less strong than in the general population, possibly due to the role of the variable mother’s weight gain, which can act as confounding factor [18]. Also, insulin resistance in pregnancy depends upon several factors, such as placental hormones and others [80], and this is another reason for lower relationship between insulin resistance and BMI during gestation. Of note, in the TyGIS formula the BW coefficient is negative, whereas that of LBM is positive. This means that higher BW determines lower TyGIS, as expected (TyGIS is a marker of insulin sensitivity). On the other side, in TyGIS formula, higher LBM determines higher TyGIS, and this again appears reasonable: since LBM is expressed as percentage of total body mass (i.e., BW), for a prescribed BW value higher LBM means lower amount of fat mass, and this is expected to determine better insulin sensitivity.

Opportunities for comparison to previous studies are limited. To our knowledge, only a couple of studies analyzed associations between TyG and insulin resistance in pregnancy [47, 51]. However, comparison of TyG to insulin resistance indices was limited to that with the fasting HOMA-IR index [81], thus in pregnancy no studies compared TyG with insulin resistance/sensitivity indices derived by dynamic tests as the OGTT, which typically reflects reference insulin sensitivity (from the clamp) remarkably better than insulin sensitivity at fasting [82–84]. In addition, in pregnancy no studies developed a version of TyG for improved relationship with insulin sensitivity from the OGTT. In Poveda’s study [47], TyG correlated with HOMA-IR in the first and second trimester of pregnancy, but not in the third trimester, and even in first and second trimesters correlation was weak. This suggests that in pregnancy TyG is not a good surrogate marker of insulin resistance, though on the other hand HOMA-IR has limits in the assessment of insulin resistance, as outlined above [82–84]. Similarly, in the Sánchez-García’s study [51] the relationship between TyG and HOMA-IR was not strong. Overall, such findings emphasize the need for an improved TyG as surrogate marker of insulin resistance (or sensitivity) in pregnancy. This is in fact what we have done in the present study developing the proposed new index, which on one side is built upon comparison with OGTT-derived insulin sensitivity rather than fasting insulin sensitivity, and on the other side it essentially preserves TyG simple formulation and hence easy applicability in the clinical practice.

Similarly to our study, in previous studies some indices based only on fasting variables were proposed as markers for insulin resistance/sensitivity [85–89]. Specifically, some studies developed an improved version of TyG, but none with pregnant women as the target population [85–87]. In terms of different fasting-based indices, one relatively popular is the triglyceride-HDL ratio (TG/HDL) [88], or its evolution (SPISE) [89]. However, after scientific literature analysis we were convinced that TyG performs somehow better than TG/HDL, and this was corroborated in our data by the slightly higher association of TyG rather than TG/HDL to PREDIM, despite such association being weak in both cases (details not shown). Thus, we developed our new index with TyG rather than TG/HDL as the starting point.

TyGIS was derived through machine learning approach. This approach is often used in large datasets, but it is also valuable in smaller datasets with many variables, as in our case [70, 90–93]. Of note, those articles [70, 90–93] emphasize the importance of the initial feature selection process, as we carried out as first step in our approach. It is also worth noting that in the specific domain of pregnancy and GDM we already applied machine learning techniques to a relatively small cohort of former GDM patients to predict the risk of developing type 2 diabetes [94]. In the machine learning approach of the current study, we first evaluated several variable subsets. Assuming the existence of non-linear dynamics, interactions between variables and their quadratic terms were also considered, but the best model was found to be a linear one. For the selection of the optimal subset, we oriented towards methods providing a simple equation, thus easy to use and hence potentially applicable in the clinical context. Therefore, SVM models were considered, with linear kernel and L2 or L1 regularization, though robust linear regression model was also tested [95]. Non-linear models were also considered, but they were not providing simple predictor coefficients, and hence did not allow to end up with an algebraic equation of easy interpretation and use [96]. The next step for final model selection was applying on the training set a nested CV procedure, this yielding unbiased performance estimates even in case of limited dataset size [70]. The selection of the optimal model was based on both the RMSE obtained from the nested CV and the BIC criteria. The final selected model was that with the lowest BIC and one of the lowest RMSE. Of note, the model with the lowest RMSE had unsatisfactory BIC, and it was in fact not able to discriminate between subgroups on the training set population (details not shown).

Our study has some limitations. The first is the size of the cohort, which was not big. However, this is justified by the wide battery of variables, including OGTT with frequent (30 min) sampling frequency rather than the traditional 1-h frequency, and with measurement of glucose, insulin and C-peptide. Such OGTT characteristics allowed reliable assessment of both insulin sensitivity and insulin secretion/beta-cell function. Indeed, both aspects were relevant for development and testing of the new index performance, since insulin sensitivity and insulin secretion/beta-cell function may be related. Thus, when testing performances of a new insulin sensitivity/resistance index, assessment of its possible relationships with insulin secretion and beta-cell function should definitely be considered. It is also worth noting that datasets larger than the dataset analyzed in the current study, with similar features (variables) available, are rare. This is likely due to the reason that investigators typically limit the burden and discomfort of the study procedures in pregnant women, due to the intrinsic frailty of the gravid condition even in the absence of any morbidity. Thus, to our knowledge few studies (one of which partly from our research group) reported datasets of pregnant women larger than the dataset under analysis with OGTT measurement of glucose, insulin and C-peptide [97–99], and at any rate those studies [97–99] missed lipid profile information (including triglycerides). In summary, we are not aware of any dataset with larger number of participants and all necessary features for the goals of the current study. On the other hand, the size of the current dataset is similar (slightly larger) to that analyzed in some of our previous studies related to pregnancy, where the glucose/insulin/C-peptide OGTT was available [56, 100–102]. Of note, in one of those studies we already applied a technique classified within the machine learning domain (i.e., principal component analysis) [101].

It also has to be reported that we searched for a precise indication about the appropriate number of samples (observations) in relation to the number of features, but we did not find a unique and robust recommendation. However, for machine learning approaches similar to ours, we found that some investigators suggested a minimum number of observations equal to the number of features plus 50 in cases up to five features, and equal to 10 observations per feature in cases of 6 or more features [103]. These indications essentially are met in our analysis, since we tested models with two to six features at most (apart for some of the models including the GDM presence variable), and in the training set we had in fact about 10 × 6 observations (precisely, 59). Moreover, if we consider the final selected model, it includes four features only. Thus, the applicable indication for the observations number is that of 50 plus the features number, yielding 54 in our case (this being fully consistent with our training set size). On the other hand, it is worth noting that the main findings of the study confirm the size of the dataset as sufficient for our purposes. Indeed, TyGIS performance was satisfactory according to all tests, in both training and test set. Furthermore, when stratifying the subjects into subgroups, both PREDIM and TyGIS consistently showed the expected differences in insulin sensitivity (i.e., higher in NGT than in non-NGT, and in lean than in overweight people). In addition, as previously outlined, results related to the relationships with model-based insulin secretion and beta-cell function parameters were typically as expected, again according to both PREDIM and TyGIS. It is also worth noting that possible larger datasets would not necessarily improve TyGIS performance further. Indeed, TyGIS by definition does not include information from metabolic tests (such as the OGTT), to maintain the original simplicity and easy use of the traditional TyG. On the other hand, it is unlikely that one index based on variables measured in non-stimulated conditions can predict with extreme accuracy the stimulated metabolic state, even in the case of very large datasets exploited for the index development. For this reason, TyGIS has to be considered a surrogate marker of insulin sensitivity, and this would hold even if TyGIS was developed on larger datasets.

Another limitation of our study was that the reference insulin sensitivity measure, as derived from a hyperinsulinemic-euglycemic clamp, was not available in our dataset. However, the OGTT data allowed calculation of PREDIM, which has same units and range of variation of the clamp-derived index, and was proved being excellent predictor of the latter [59]. Nonetheless, another issue to consider is whether PREDIM was validated in a sufficiently wide range of insulin sensitivity values, thus comprising values typical of insulin resistance observable in pregnancy, especially when complicated by GDM. To this purpose, it is convenient examining the typical values of insulin sensitivity in pregnancy, as assessed by the reference clamp-derived index. One study reported the clamp *M* parameter in both GDM and non-GDM pregnant women, in each trimester of pregnancy [104]. Lowest average values were observed in GDM in the third trimester (2.70 ± 0.81 mg∙kg^−1^∙min^−1^, mean ± SD). This translates into 95% interval (mean ± 2 × SD) equal to 1.08–4.32 mg∙kg^−1^∙min^−1^. However, although 1.08 mg∙kg^−1^∙min^−1^ is quite low insulin sensitivity value, PREDIM validation included such low values (lowest values ≈0.5 mg∙kg^−1^∙min^−1^ [59]). Other studies reported in pregnancy similar clamp *M* parameter values [105, 106]. On the other hand, it should be acknowledged that PREDIM formula includes the BMI, and such variable may be not completely reliable in pregnancy. However, based on our analyses, this potential bias may determine inaccuracies in PREDIM calculation not exceeding 10–15%, which appears acceptable from a clinical point of view (details not shown). It is also worth noting that PREDIM provided reliable findings in some previous studies in pregnancy [56, 102, 107].

One further limitation was that the lean body mass, which resulted as feature selected for the final TyGIS formula, was not directly measured, but estimated by an empirical formula [67]. However, the formula is widely accepted and has been extensively used in many investigations. In addition, though not specific for pregnancy, where a contribution to the lean body mass may come from the fetus, the used empirical formula is specific for females, thus we assumed it as sufficiently reliable.

Finally, it is necessary to recommend some restrictions in the use of TyGIS. Indeed, despite TyGIS being remarkably improved compared to the traditional TyG as predictor of insulin sensitivity/resistance, it remains a surrogate marker. Thus, TyGIS is appropriate for possible comparison among different subgroups of pregnant women, but the investigator should be aware that the value in a single subject may be affected by a relevant error. As a fact, whenever possible, insulin sensitivity/resistance should be always assessed stimulating subject’s metabolism with glucose or food administration, as with the OGTT. On the other hand, when the metabolic test is not practically feasible (such as in possible epidemiological studies including large cohorts), TyGIS has proven as good insulin sensitivity surrogate marker, at least in pregnancy.

It may also be asked whether the TyGIS formula could be applied to other populations. In our opinion, even in different populations TyGIS may perform better than TyG, as marker for insulin sensitivity/resistance. However, this was not proved in the present study, thus we do not recommend TyGIS use in other populations. Nonetheless, the current study may trigger future studies aimed at developing improved TyG-based surrogates of insulin sensitivity/resistance specifically tailored for different populations, in the context of precision medicine [108–111]. In such new studies, the methodology of the present study may be exploited as guide for a robust approach. On the other hand, in pregnancy future studies may explore the possible TyGIS ability to predict relevant aspects other than insulin sensitivity, such as maternal/neonatal outcomes.

## Conclusions

We developed an improved version of the triglyceride-glucose index (TyG), as a new surrogate marker of insulin sensitivity in pregnancy. Similarly to the traditional TyG, the new index, called TyGIS, does not require OGTT or other metabolic tests, but its performances as surrogate marker of insulin sensitivity are remarkably improved than those of TyG. Thus, although the stimulation of metabolism with a glucose load or a meal (as in the OGTT or meal test) remains preferable for insulin sensitivity assessment, when such metabolic test is not possible for any reason, in populations of pregnant women the use of TyGIS as marker of insulin sensitivity appears acceptable.

## Supplementary Information

**Additional file 1: Table S1.** Subsets obtained from the feature selection procedure (from 2 to 5, as no subset was selected with 6 features).

## Data Availability

The datasets generated and analyzed during the current study are not publicly available due privacy policy issues, but they are available from the corresponding author on reasonable request.

## Abbreviations

ALAT: Alanine aminotransferase
ANOVA: Analysis of variance
ASAT: Aspartate aminotransferase
BIC: Bayesian information criterion
BMI: Body mass index
BSA: Body surface area
BSR: Basal insulin secretion rate
BW: Body weight
C.V.: Coefficient of variation
CI: Confidence interval
CV: Cross-validation
DCCT: Diabetes control and complications trial
GDM: Gestational diabetes mellitus
GGT: Gamma glutamyltransferase
G-sens: Glucose sensitivity
Hb: Hemoglobin
HbA1c: Glycated hemoglobin
HDL: High-density lipoprotein
HIV: Human immunodeficiency virus
HOMA-IR: Homeostatic model assessment for insulin resistance
IFCC: International Federation of Clinical Chemistry
IQR: Interquartile range
LASSO: Least absolute shrinkage and selection operator
LBM: Lean body mass
LDL: Low-density lipoprotein
LGA: Large for gestational age
MSE: Mean squared error
NCA: Neighborhood component analysis
NGT: Normal glucose tolerance
OGTT: Oral glucose tolerance test
OR: Odds ratio
PFR: Potentiation factor
PREDIM: Predicted M
RF: Random forest
RMSE: Root mean squared error
R-sens: Rate sensitivity
RT: Regression tree
SPISE: Single point insulin sensitivity estimator
SVM: Support vector machine
TG: Triglycerides
TIS: Total insulin secretion
TyG: Triglyceride-glucose index
TyGIS: TyG for insulin sensitivity
