# Supplementary material for: TyGIS: improved triglyceride-glucose index for the assessment of insulin sensitivity during pregnancy
Source: Cardiovasc Diabetol. 2022 Oct 18;21:215. doi: 10.1186/s12933-022-01649-8 (PMC9580191; doi:10.1186/s12933-022-01649-8)
Supplement: Supplementary file 1 — Additional file 1: Table S1. Subsets obtained from the feature selection procedure (from 2 to 5, as no subset was selected with 6 features). [file 12933_2022_1649_MOESM1_ESM.pdf]

Appendix A - TyGIS: Improved triglyceride-glucose index for the assessment of insulin sensitivity during pregnancy - Details on the machine learning approach

Feature selection: A careful feature selection phase was carried out to reduce possible overfitting determined by excessive features to sample size ratio [1]. Feature selection was based only on the training set to avoid the information leakage that would result from inclusion of the entire dataset in this phase [1]. Different possible feature subsets were evaluated to be used as inputs to the following model formulation steps. Such subsets were selected through the following approaches: *(i)* Feature ranking in terms of the relevance of features with respect to the output (*i.e.*, the insulin sensitivity index, PREDIM [2]) was obtained based on two procedures, *i.e.*, the F-test [3] performed on all features, and the diagonal adaptation of neighborhood component analysis (NCA), performed on continuous features only [4]. Starting from the results from each procedure, the subsets containing the *n* most important features were considered, with *n* from 1 to 5. *(ii)* Sequential feature selection based on minimization of mean squared error (MSE) loss function [5], considering only the continuous features and with the constraint of inclusion among predictors of the traditional triglyceride-glucose index (TyG) [6]. Loss functions of several machine learning models were minimized, namely, Regression Tree (RT) [7], an ensemble of RTs [8], Random Forest (RF) [9], Support Vector Machine (SVM) with linear kernel, SVM with radial kernel, SVM with polynomial grade 2 kernel [10]. Constraints were imposed on the number of features to be included, varying from 2 to 6. These models were run with the MATLAB functions default settings, applying 10-fold cross-validation (CV) for the calculation of the MSE. This phase of the procedure yielded the selection of 17 subsets (see Table A.1). Furthermore, indicating with x_i the *i-th* subset feature and with p the number of subset features, in addition to the model based on the original subset $\{x_i, with\ i\ from\ 1\ to\ p\}$, in some cases we also considered the following models: the original subset plus the interactions between the features $\{x_i,\ with\ i\ from\ 1\ to\ p\} \cup \{x_i x_j, with\ i, j\ from\ 1\ to\ p, i \neq j\}$, and the original subset plus the indicated interactions and the quadratic terms of the features $\{x_i, i\ from\ 1\ to\ p\} \cup \{x_i x_j, with\ i, j\ from\ 1\ to\ p\}$. Precisely, we considered interactions and quadratic terms for the subset with two features only (see Table A.1), and interactions (but no quadratic terms) for the four subsets with three features each. Neither interactions nor quadratic terms were considered for the other twelve

subsets (*i.e.*, only the original subset). This led to 1 × 3 + 4 × 2 + 12 = 23 subsets. Each subset was then also considered with the addition of the dichotomous gestational diabetes mellitus (GDM) variable, hypothesized as possibly relevant variable for insulin sensitivity prediction, leading to 46 subsets as inputs for the following phases in model formulation.

Final model: By the L2-regularized SVM method [11] we built a prediction model with regularization term within the loss function, whose weight was determined by the λ hyperparameter:

$$\sum_{i=1}^{N} max[0, |y - \hat{y}| - \varepsilon] + \frac{\lambda}{2} \sum_{j=1}^{P} \beta_j^2 \quad \text{(A.1)}$$

N represents the number of training examples, y and $\hat{y}$ the true and the predicted insulin sensitivity index, respectively, ε the width of the error-insensitive band, *p* the number of inputs to the model, and β_j the *j-th* input estimated coefficients [12]. For the estimation of the model parameters and hyperparameter, a nested CV technique was implemented, this producing solid and unbiased performance estimates even with small datasets [1]. The nested CV procedure consisted of an outer and an inner loop. In the inner loop a 5-fold CV was used to tune the hyperparameter λ: precisely, a Bayesian optimization method was implemented [13]. The outer loop implemented the K-fold CV method (here, with K=10) [14], where the training set was divided into K folds and the training phase repeated K times, each of which considering K-1 folds as the training set and the remaining fold as the validation set. At each step, the root mean squared error was calculated on each validation fold ($RMSE_{ik}$ in Figure 1 of the main text), and the average $RMSE_i$ over the 10 steps of the procedure was then obtained. The procedure was repeated for each of the 46 different inputs to the model, the best inputs being assumed as those with lower average $RMSE_i$. However, for ensuring low features to sample size ratio [1], in the selection of the final model both the $RMSE_i$ and the Bayesian information criterion (BIC) [15] values were considered (see main text).

Table A.1: Subsets obtained from the feature selection procedure (from 2 to 5, as no subset was selected with 6 features). Subsets are presented for increasing number of features. For a given number of features, subsets are alphabetically ordered, and the same holds for the features in

each subset. The subset finally selected for the new index formulation (TyGIS) is marked in bold. BMI: body mass index; BSA: body surface area; LBM: lean body mass.

**Subset sequential order**	**Feature 1**	**Feature 2**	**Feature 3**	**Feature 4**	**Feature 5**
SUBSET1	Fasting Insulin	TyG			
SUBSET2	Age	Fasting Insulin	TyG		
SUBSET3	BMI	Fasting Insulin	TyG		
SUBSET4	Body Weight	Fasting Insulin	TyG		
SUBSET5	Fasting C-peptide	Fasting Insulin	TyG		
SUBSET6	Age	BMI	Fasting Insulin	TyG	
SUBSET7	Age	Creatinine	Fasting Insulin	TyG	
SUBSET8	BMI	Body Weight	Fasting Insulin	TyG	
SUBSET9	BMI	Creatinine	Fasting Insulin	TyG	
SUBSET10	BMI	Fasting Glucose	Fasting Insulin	TyG	
SUBSET11	BMI	Fasting Insulin	Height	TyG	
SUBSET12	BMI	Fasting Insulin	LBM	TyG	
**SUBSET13**	**Body Weight**	**Fasting Insulin**	**LBM**	**TyG**	
SUBSET14	Age	Creatinine	Fasting Insulin	Total pregnancy number	TyG
SUBSET15	BMI	BSA	Fasting Insulin	Height	TyG
SUBSET16	BMI	Creatinine	Fasting Insulin	Hemoglobin	TyG
SUBSET17	BMI	Body Weight	Fasting C-peptide	Fasting Insulin	TyG

References

1. Vabalas A, Gowen E, Poliakoff E, Casson AJ. Machine learning algorithm validation with a limited sample size. Hernandez-Lemus E, editor. PloS One. 2019;14:e0224365.

2. Tura A, Chemello G, Szendroedi J, Göbl C, Færch K, Vrbíková J, et al. Prediction of clamp-derived insulin sensitivity from the oral glucose insulin sensitivity index. Diabetologia. 2018;61:1135–41.

3. Univariate feature ranking for regression using F-tests - MATLAB fsrftest - MathWorks Italia [Internet]. 2022 [cited 2022 Jun 15]. Available from: https://it.mathworks.com/help/stats/fsrftest.html

4. Feature selection using neighborhood component analysis for regression - MATLAB fsrnca - MathWorks Italia [Internet]. 2022 [cited 2022 Jun 15]. Available from: https://it.mathworks.com/help/stats/fsrnca.html

5. Aha DW, Bankert RL. A Comparative Evaluation of Sequential Feature Selection Algorithms. In: Fisher D, Lenz H-J, editors. Learn Data [Internet]. New York, NY: Springer New York; 1996 [cited 2022 Jun 6]. p. 199–206. Available from: http://link.springer.com/10.1007/978-1-4612-2404-4_19

6. Simental-Mendía LE, Rodríguez-Morán M, Guerrero-Romero F. The Product of Fasting Glucose and Triglycerides As Surrogate for Identifying Insulin Resistance in Apparently Healthy Subjects. Metab Syndr Relat Disord. 2008;6:299–304.

7. Breiman L, Friedman JH, Olshen RA, Stone CJ. Classification And Regression Trees [Internet]. 1st ed. Routledge; 2017 [cited 2022 Jun 6]. Available from: https://www.taylorfrancis.com/books/9781351460491

8. Breiman L. Bagging predictors. Bagging Predict. 1996;24:123–40.

9. Breiman L. Random forests. Mach Learn. 2001;45:5–32.

10. Gunn SR. Support vector machines for classification and regression. Technical Report, School of Electronics and Computer Science, University of Southampton. 1998.

11. Chang Y-W, Hsieh C-J, Chang K-W, Ringgaard M, Lin C-J. Training and Testing Low-degree Polynomial Data Mappings via Linear SVM. J Mach Learn. 2010;11:1471–90.

12. Fit linear regression model to high-dimensional data - MATLAB fitrlinear - MathWorks Italia [Internet]. 2022 [cited 2022 Jun 15]. Available from:

https://it.mathworks.com/help/stats/fitrlinear.html?searchHighlight=fitrlinear&s_tid=srchtitle_fitrlinear_1

13. Snoek J, Larochelle H, Adams RP. Practical Bayesian Optimization of Machine Learning Algorithms. arXiv; 2012 [cited 2022 Jun 6]; Available from: https://arxiv.org/abs/1206.2944
14. Fushiki T. Estimation of prediction error by using K-fold cross-validation. Stat Comput. 2011;21:137–46.
15. Neath AA, Cavanaugh JE. The Bayesian information criterion: background, derivation, and applications. WIREs Comput Stat. 2012;4:199–203.
